# Network analysis of hepatocellular carcinoma liquid biopsies augmented by single-cell sequencing data

**DOI:** 10.3389/fgene.2022.921195

**Published:** 2022-08-25

**Authors:** Aram Safrastyan, Damian Wollny

**Affiliations:** ^1^ RNA Bioinformatics and High Throughput Analysis, Friedrich Schiller University Jena, Jena, Germany; ^2^ Leibniz Institute on Aging-Fritz Lipmann Institute (FLI), Jena, Germany; ^3^ Max Planck Institute for Evolutionary Anthropology, Leipzig, Germany

**Keywords:** hepatocellular carcinoma, liquid biopsy, WGCNA, cell-free RNA, single cell sequencing

## Abstract

Liquid biopsy, the analysis of body fluids, represents a promising approach for disease diagnosis and prognosis with minimal intervention. Sequencing cell-free RNA derived from liquid biopsies has been very promising for the diagnosis of several diseases. Cancer research, in particular, has emerged as a prominent candidate since early diagnosis has been shown to be a critical determinant of disease prognosis. Although high-throughput analysis of liquid biopsies has uncovered many differentially expressed genes in the context of cancer, the functional connection between these genes is not investigated in depth. An important approach to remedy this issue is the construction of gene networks which describes the correlation patterns between different genes, thereby allowing to infer their functional organization. In this study, we aimed at characterizing extracellular transcriptome gene networks of hepatocellular carcinoma patients compared to healthy controls. Our analysis revealed a number of genes previously associated with hepatocellular carcinoma and uncovered their association network in the blood. Our study thus demonstrates the feasibility of performing gene co-expression network analysis from cell-free RNA data and its utility in studying hepatocellular carcinoma. Furthermore, we augmented cell-free RNA network analysis with single-cell RNA sequencing data which enables the contextualization of the identified network modules with cell-type specific transcriptomes from the liver.

## Introduction

Hepatocellular carcinoma (HCC) is the most common form of primary liver cancer accounting for almost 700,000 deaths worldwide annually, making it one of the leading causes of cancer-related deaths worldwide (Patel et al., 2015; Black and Mehta, 2018). Treatment of HCC represents a challenge due to late diagnosis of the disease (Patel et al., 2015) further increasing the need to improve diagnostic methods. As direct tissue sampling from the liver is hard to conduct, less invasive procedures are needed. Liquid biopsy represents a promising alternative to invasive tissue biopsies in particular for cancer diagnosis as it enables the study of cell-free (cf) nucleic acids in the blood, which includes cfDNA and various cfRNAs (e.g., protein-coding, lncRNA, microRNA, etc.). Since the sources of the extracellular transcriptome are not only blood cells, liquid biopsies represent a way to gain insights into gene expression changes in solid tissues without the need for surgical intervention (Vorperian et al., 2022). In fact, a number of HCC liquid biopsy studies have already been conducted indicating the potential of this approach for HCC diagnostics (Qu et al., 2019; Labgaa et al., 2021; Zhu et al., 2021; Yang et al., 2022).

An underexplored way of analyzing the rich data obtained from sequencing liquid biopsy-derived cfRNA liquid biopsy is weighted gene co-expression network analysis (WGCNA). WGCNA tries to find genes that have correlated expressions and aims to build sets of such genes which are named modules. This correlation indicates that linked genes are likely part of a shared regulatory mechanism in the cell. In comparison to the frequently used differential gene expression analysis the approach offers additional insights by incorporating information about the relationship between genes detected in a given sample. The study of genes present in a module can also generate hypotheses about the function of a previously undescribed gene *via* the concept of “guilt by association”. Finally, modules can be correlated with phenotype traits and by further studying the module with a high correlation with a trait of interest gain a better understanding of the molecular underpinnings of the trait itself.

To date, many studies have focused on WGCNA analysis of bulk or single-cell RNA (scRNA) sequencing data from tissue samples. However, WGCNA is only beginning to be applied to cfRNA data. Hence, we performed WGCNA analysis on cfRNA data derived from the blood of HCC patients. We have found cfRNA-derived network modules which strongly correlate with the disease trait. Pathway analysis was performed to gain further insights into the biological functions that are associated with the discovered modules. We further validate the robustness of our findings in an independent cfRNA dataset from HCC patients. Lastly, we leveraged single-cell RNA sequencing data to gain insights into potential cellular sources of cfRNA network modules.

## Materials and methods

### Data preprocessing of cell-free RNA datasets

The liquid biopsy datasets were generated by Zhu et al. (2021), Li et al. (2018) and were obtained from NCBI Gene Expression Omnibus (GEO) under accession numbers GSE142987 and GSE100207 respectively (Table 1). The dataset GSE142987 contained RNA sequencing data of plasma cell-free total RNA (cfRNA) from 30 healthy donors and 35 hepatocellular carcinoma patients (Table 1). The detailed steps of sample collection, processing and data analysis are described in the corresponding paper (Zhu et al., 2021). The final count matrix which served as input for further analysis containing raw reads of all transcripts not mapping to the rRNA database was deposited in GEO. After acquiring the count matrix, the data was prepared for further analysis in WGCNA. To that end, the read count matrix and the accompanying metadata file were used to construct a DESeqDataSet object with the function “DESeqDataSetFromMatrix” from R (version 4.1.1) (R Core Team, 2021) package DESeq2 (version 1.34.0) (Love et al., 2014). Genes with fewer than five normalized reads in 10% of the samples were filtered out. The data was variance stabilized (as recommended in WGCNA FAQ) and exported with the function “getVarianceStabilizedData” from the package DESeq2. The final dataset consisted of 9,200 genes and 65 samples.

**TABLE 1:** Overview of the RNA sequencing datasets used in the current study.

Record	Data source	Platform	Healthy samples	HCC samples	References
GSE142987	Blood (cfRNA)	Illumina HiSeq X Ten	30	35	Zhu et al. (2021)
GSE100207	Blood (exoRNA)	Illumina HiSeq 2000	—	21	Li et al. (2018)
GSE115469	Liver (scRNA)	Illumina HiSeq 2500	5 (8,444 cells)	—	MacParland et al. (2018)

The second cfRNA dataset generated by Li et al. (2018) contained RNA sequencing data of blood exosomal total RNA (exoRNA) from 21 hepatocellular cancer patients (Table 1). The detailed steps of sample collection and processing are detailed in the corresponding paper. The raw sequencing files (.fastq.gz) were deposited in GEO and were downloaded using the tool “fasterq-dump” from the NCBI SRA-Toolkit (version 2.11.0) (SRA Toolkit Development Team, 2022). Next, the sample files were mapped to the human reference genome (build GRCh38) with STAR package (version 2.7.8a) (Dobin et al., 2013) using the default parameters. The read count matrix was generated by utilizing the function “featurecounts” from the Subread package (version 2.0.1) (Liao et al., 2014) with the parameters −p and −s 1. Next, the data was imported into DESeq2, filtered as already described and exported after variance stabilization. The final dataset contained 11,485 genes and 21 samples.

### Data preprocessing of the single-cell RNA dataset

The single-cell RNA (scRNA) sequencing of liver tissue biopsies was carried out by MacParland et al. (2018) and deposited in GEO under the accession number GSE115469 (Table 1) and contained quality filtered cell-type annotated single-cell sequencing data containing 8,444 cells obtained from five healthy human liver donors. The dataset was used as input for the R (version 4.1.2) package Seurat (version 4.1.0) (Hao et al., 2021). Subtypes of hepatocyte, T cell, liver sinusoidal endothelial cell (LSEC) and macrophage were aggregated for each cell type respectively in order to capture the most complete transcriptomic landscape of each cell type. Next, the data was normalized and scaled using the Seurat functions “NormalizeData” and “ScaleData” respectively. UMAP dimensional reduction was run with the Seurat function “RunUMAP” using the 5,000 most variable genes. Afterwards, the data was prepared for analysis with WGCNA. As WGCNA was originally developed for the analysis of bulk RNA sequencing data, the scRNA sequencing data was transformed for single-cell WGCNA analysis as previously described (Morabito et al., 2021). Aggregation was carried out on a per cell type basis using the “construct_metacells” function from the R package scWGCNA (version 0.0.0.9000) (Morabito et al., 2021). Hepatic stellate cells were excluded from this step and further downstream analysis due to very low number of cells. The k parameter of the “construct_metacells” function was set according to the number of cells that each cell type contained: eight in the case of fewer than 300 cells and 20 otherwise. Thus, on average less than 10% of cells were shared between paired metacell constructs.

In the end, the transformed data consisted of 20,007 genes and 5,266 cells corresponding to 10 cell types: LSECs (355), NK-like cells (291), cholangiocytes (101), erythroid cells (71), hepatocytes (2,230), macrophages (704), mature B cells (105), plasma cells (296), portal endothelial cells (178) and T cells (935). The final data was normalized, scaled and used for UMAP dimensionality reduction as already described. Final plots were generated using the function “DimPlot” from the package Seurat and the R package ggplot2 (version 3.3.6) (Hadley, 2016).

### Gene co-expression network construction

Variance stabilized cfRNA dataset (Zhu et al.) was used as input for WGCNA (version 1.69) (Langfelder and Horvath, 2008). Outlier samples were detected by using the base R stats package function “hclust” with method = “average” and WGCNA function “cutTreestatic” with parameters cutHeight = 100 and minSize = 2. This resulted in the removal of six samples (three healthy and three cancer). Ensembl gene IDs were converted to HGNC (HUGO Gene Nomenclature Committee) symbols using the function “getBM” from R package biomaRt (version 2.50.0) (Durinck et al., 2005). A final matrix of 59 samples and 9,038 genes was used for network construction. Here, to pick a suitable power for WGCNA analysis the function “pickSoftThreshold” was used from the package WGCNA with parameter networkType = “unsigned”. Based on the derived plots generated by R package ggplot2 (Supplementary Figure S1A) and the recommendations from the WGCNA manual a power of six was chosen here where it achieved both scale-free topology and a suitable number of mean connectivity. The construction of modules was comprised of the following steps:

• Building an adjacency matrix using the function “adjacency” from the package WGCNA with parameters power = 6 and type = “unsigned”,
• Building a Topological Overlap Matrix (TOM) using the function “TOMsimilairty” from the package WGCNA with parameter TomType = “unsigned”,
• Performing hierarchical clustering using the function “hclust” from the R base package stats with parameter method = “average”,
• Identification of modules using the function “cutreeDynamic” from the package dynamicTreeCut (version 1.63-1) (Langfelder et al., 2008) with parameter minClusterSize = 30 and deepSplit = 2,
• Numeric labels were converted into colors using the function labels2colors from the package WGCNA and similar modules were merged using the function “mergeCloseModules” from the package WGCNA with parameter cutHeight = 0.2.

For the exoRNA dataset (Li et al.) no outlier samples were detected. The same steps were conducted as already described with the exception of the parameter: power = 8 (Supplementary Figure S1B). Plots describing the number of genes in cfRNA and exoRNA modules were generated using the R package ggplot2.

Gene connectivity per module, defined as the sum of the adjacency to other genes of the same module, was calculated using the WGCNA (version 1.71) function “softConnectivity” with the parameter power set to six for cfRNA and eight for exoRNA datasets. Top 50 most connected genes per module are displayed in Supplementary Material S1 (cfRNA) and Supplementary Material S2 (exoRNA).

### Module-trait associations

To calculate the module associations with sample traits, first cfRNA module eigengenes were computed with the function “moduleEigengenes” from the package WGCNA. Then Pearson correlation values were computed between module eigengenes and sample traits (“age,” “disease_state,” and “gender”) with the WGCNA function “cor.” In the case of “disease_state” trait, healthy samples were denoted as “0” and cancer samples as “1”. For the trait “gender” male samples were denoted as “0”, while female samples as “1”. Student asymptotic *p*-values were calculated with the function “corPvalueStudent” from the package WGCNA and corrected for multiple testing using the base R function “p.adjust” with method = “fdr” (Benjamini and Hochberg, 1995). Finally, a heatmap was constructed using the R package ggplot2. Top hub genes were detected by using the WGCNA function “chooseTopHubInEachModule” with parameters power = 6 and type = “unsigned”.

In the exoRNA dataset as all the samples originated from HCC patients, module-trait association was not performed. In the cfRNA dataset, the grey module which contains unassigned genes was removed from further analysis with the WGCNA function “removeGreyME”.

### Module membership vs. gene-trait significance analysis

To generate scatterplots of cfRNA module membership and gene-trait significance correlation, module membership which describes the closeness of genes to individual modules, was calculated by conducting a Pearson correlation of gene expression values with module eigengenes with WGCNA function “cor”. Gene-trait significance was calculated by conducting a Pearson correlation between gene expression values and the trait “disease_state” with the function “cor” from the package WGCNA. Scatterplots were generated with the R package ggplot2.

### Module preservation analysis

To calculate the preservation statistics of cfRNA modules (reference data) in the exoRNA dataset (test data) the function “modulePreservation” from the package WGCNA was employed. The function by random permutation of module assignments in the test data calculates a composite Zsum statistic which itself is composed of Zconnectivity and Zdensity statistics, denoting the preservation of interconnectedness of module nodes and preservation of module connectivity patterns. A detailed description of the method is available in the corresponding paper (Langfelder et al., 2011). The function was used with the parameters nPermutations = 100, randomSeed = 1, quickcor = 0, maxModuleSize = 5,000, and maxGoldModuleSize = 5,000 where the value 5,000 was chosen based on the largest module of the reference data. The same steps were taken to calculate module preservation statistics of exoRNA modules in cfRNA dataset with maxModuleSize = 5,500 and maxGoldModuleSize = 5,500. The grey (unassigned genes) and gold (random sample of network genes) modules were left out of the visualization. The results were visualized with the R package ggplot2. For better visualization, values were pseudo-log transformed.

For module preservation analysis of cfRNA modules in cell-type specific scRNA datasets, the WGCNA function “modulePreservation” was employed as already described with maxModuleSize and maxGoldModuleSize set to 5,000. The analysis was conducted with 5,000 most variable genes of each cell-type specific dataset which were computed as already described. The grey and gold modules were left out of the visualization since they are not informative as described above. Same steps were taken to calculate the preservation of exoRNA modules in cell-type specific scRNA datasets with the parameters maxModuleSize = 5,500 and maxGoldModuleSize = 5,500. Results were visualized using R package ggplot2.

### Module visualization

In order to visualize the identified modules, the function “exportNetworkToCytoscape” from package WGCNA was used with parameter threshold = 0.1 to export the modules consisting of 30 genes with the highest intramodular connectivity and filter out genes with adjacency threshold smaller than 0.1. Next, the exported modules were visualized with the software Cytoscape (version 3.9.1) (Shannon et al., 2003). In Cytoscape the degree of opacity of connections between genes (nodes) was set according to the weights of the connections (edges) as calculated by WGCNA. The top hub genes of each module, which were generated as already described, were also highlighted.

### Functional pathway enrichment analysis

Finally, the genes of cfRNA and exoRNA modules (cf-blue, cf-turquoise, cf-purple, cf-yellow, exo-brown) were used for Reactome (a manually curated database of functional pathways (Gillespie et al., 2022)) pathway enrichment analysis with the R (version 4.1.2) package ReactomePA (version 1.38.0) (Yu and He, 2016). For compatibility with the ReactomePA package, gene symbols were converted to Entrez gene IDs with the function “select” from the R package AnnotationDbi (version 1.54.1) (Pagès et al.). The enrichment analysis was carried out by using the ReactomePA function “enrichPathway” with parameters readable = “T” and pvalueCutoff = 0.05.

Pathway enrichment analysis of the same cfRNA and exoRNA modules was also carried out with the KEGG (Kyoto Encyclopedia of Genes and Genomes (Kanehisa et al., 2021)) and WikiPathways (Martens et al., 2021) databases using the functions “enrichKEGG” and “enrichWP” of R package clusterProfiler (version 4.2.2) (Wu et al., 2021) respectively. The results were visualized with the R package ggplot2.

For all cfRNA and exoRNA modules pathway enrichment analysis was carried out using the “Biological Process” ontology of GO (Gene Ontology) database (Gene Ontology Consortium, 2021) with the function “enrichGO” from the R package clusterProfiler where the parameter “ont” was set to “BP.” Up to five most significant results are displayed in Supplementary Material S3 and Supplementary Material S4 for cfRNA and exoRNA modules respectively.

## Results

### Construction and visualization of weighted gene co-expression network analysis modules from hepatocellular carcinoma cfRNA

We performed WGCNA analysis, in order to probe the gene co-expression network of extracellular transcriptomes from the blood of healthy control donors as well as HCC patients. The analysis resulted in the identification of eight modules describing gene networks (Supplementary Figure S2A). The resulting modules varied greatly in size, containing between 158 (cf-purple module) and 4,620 (cf-turquoise module) genes (Supplementary Figure S2A).

**FIGURE 1:**
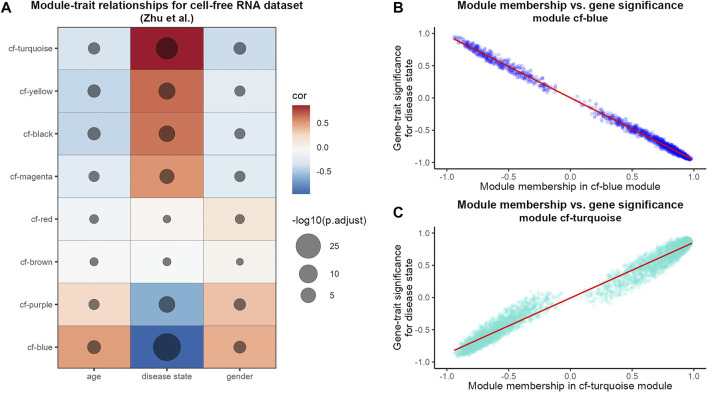
WGCNA analysis of HCC cfRNA sequencing data. **(A)** Pearson correlation results of module eigengenes and sample traits. The color scale reflects the strength of the correlation and the size of the point is proportional to the −log10 transformed corrected *p*-values. Modules are sorted based on correlation strength with the trait “disease state.” Scatterplot of cfRNA modules cf-blue **(B)** and cf-turquoise **(C)** gene module membership and gene significance for the trait “disease state.” Gene module membership refers to Pearson correlation of gene expression values and module eigengene; gene significance for the trait “disease state” refers to Pearson correlation of gene expression values and sample trait “disease state”. The red line refers to linear regression fit.

In order to test which of the derived modules correlates with patient traits, we performed a module-trait association analysis of cfRNA modules (Figure 1A). While using the age, gender and disease state (cancer vs. healthy) as traits, we found that half of the detected modules show a strong and significant positive correlation with the disease state while two modules show a significant negative correlation (Figure 1A). Next, we further examined the two modules which displayed the highest (positive and negative) correlation with the disease state of the patient in detail. When we examined the relationship between how strongly a gene is connected to a module (module membership) and its strength of correlation with the disease state (Figures 1B,C), we saw a very high correlation for both, the cf-blue as well as the cf-turquoise module. This indicates that for example in the case of the cf-turquoise module, genes with a high module membership are also very strongly associated with HCC in the blood of patients.

**FIGURE 2:**
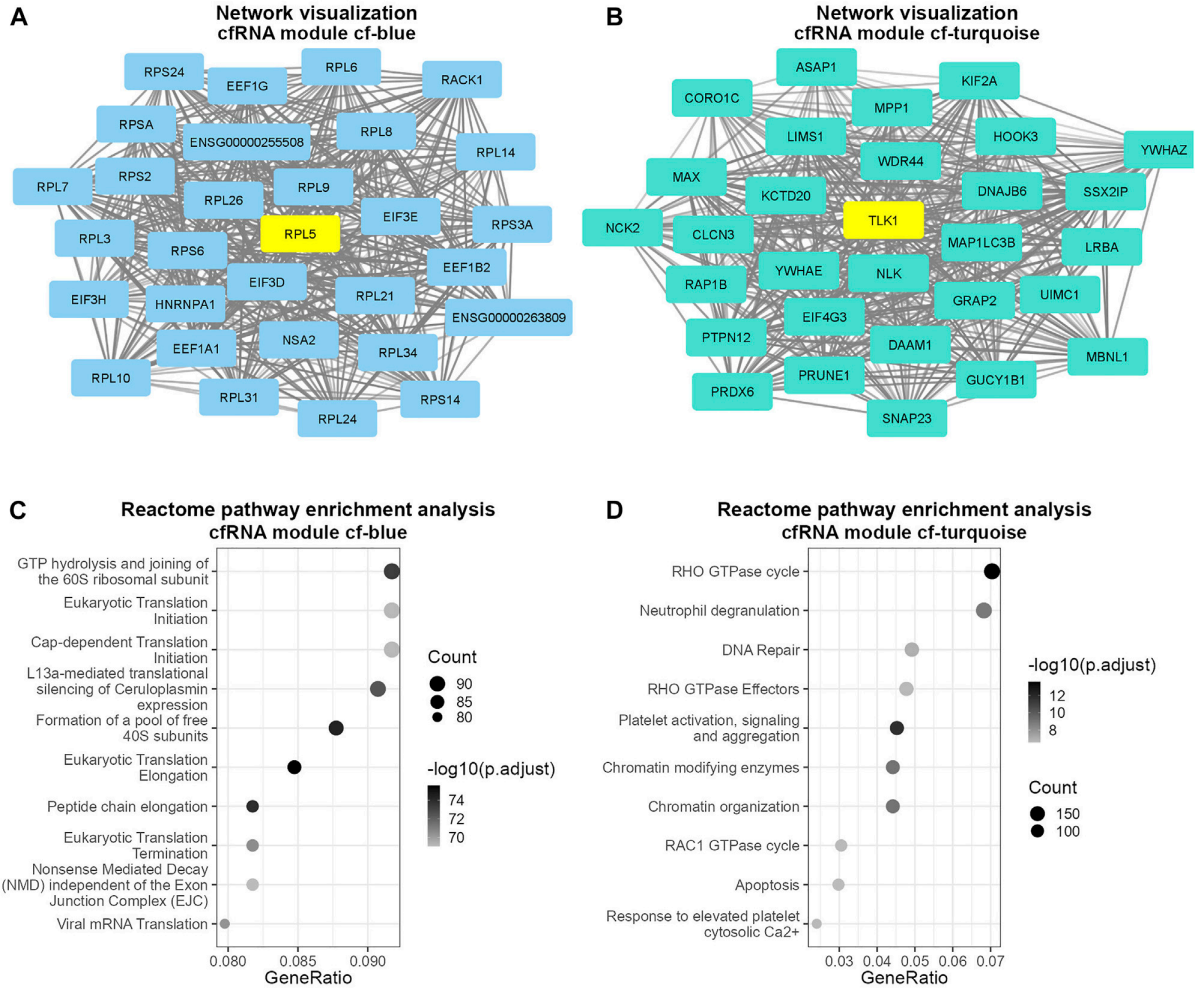
Visualization and pathway enrichment analysis of cfRNA modules cf-blue and cf-turquoise. Visualization of 30 most connected genes in modules cf-blue **(A)** and cf-turquoise **(B)**; degree of the opacity of connections is proportional to the weight of the connections; top hub gene of each module is colored in yellow. Dot plot of pathway enrichment analysis results of cfRNA modules cf-blue **(C)** and cf-turquoise **(D)**; point size denotes the number of genes in each pathway; the color scale is proportional to the −log10 transformed adjusted *p*-value; GeneRatio describes the proportion of genes found in each pathway relative to the total number of input genes found in the database.

Next, we investigated which biological pathways the cf-blue and cf-turquoise modules are associated with. The cf-blue module was enriched for pathways involved in gene translation (Figures 2A,C, Supplementary Figures S3A,B). In contrast the cf-turquoise module displays, besides enrichment for RHO GTPase activity, pathways related to immune cell activation such as neutrophil degranulation or platelet activation (Figures 2B,D, Supplementary Figures S3C,D).

### Strong module preservation across datasets

**FIGURE 3:**
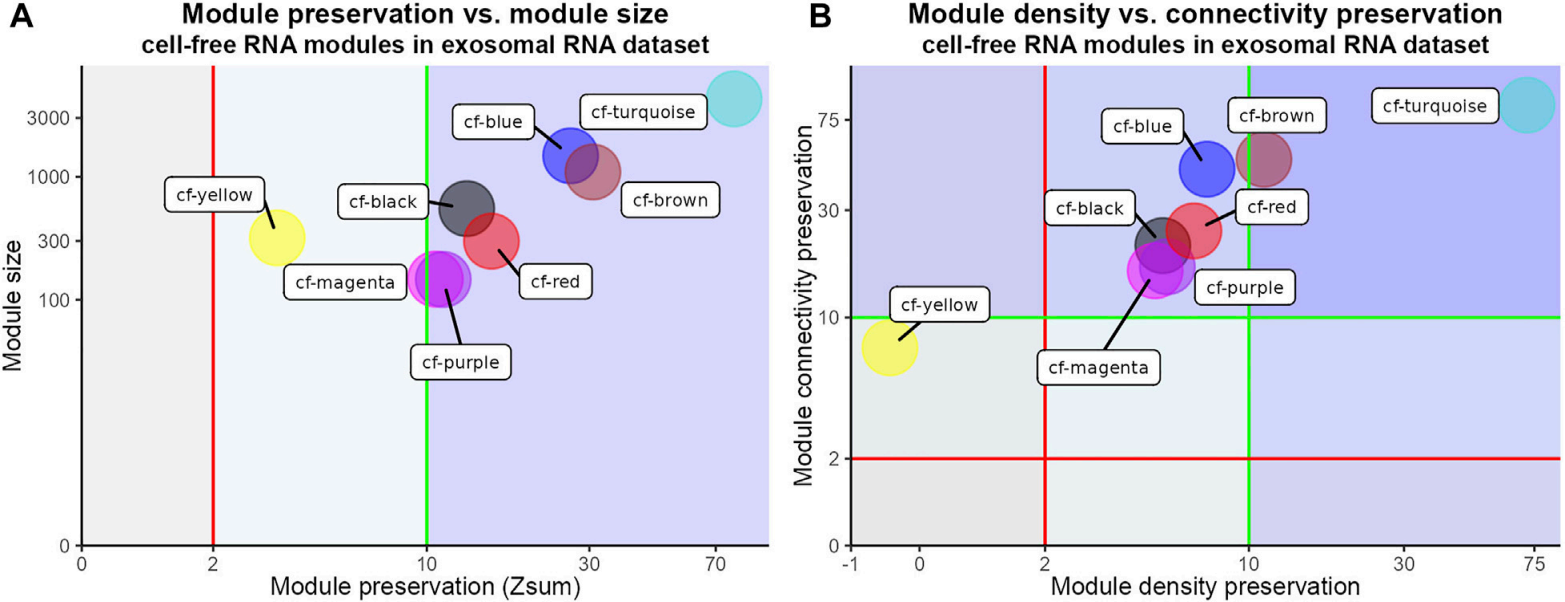
Module preservation analysis of cfRNA modules in exoRNA dataset. **(A)** Scatter plot of overall preservation statistic (Zsum) of cfRNA modules in exoRNA dataset and cfRNA module sizes. Red and green vertical lines represent weak to moderate (Zsum > 2) and strong (Zsum > 10) evidence of module preservation respectively. Axes have been pseudo-log transformed. **(B)** Scatter plot of density (Zdensity) and connectivity (Zconnectivity) preservation of cfRNA modules in exoRNA dataset. Red and green vertical lines represent weak to moderate (Zdensity > 2) and strong (Zdensity > 10) evidence of module density preservation. Axes have been pseudo-log transformed. Red and green horizontal lines represent weak to moderate (Zconnectivity > 2) and strong (Zconnectivity > 10) evidence of module connectivity preservation.

Next, we wanted to test whether and to what degree the modules identified in the cfRNA dataset (reference network) were reproducible in another liquid biopsy (test network). Hence, we performed a module preservation analysis of the cfRNA modules in an independent dataset (Li et al., 2018). In this dataset, RNA from blood exosomes derived from HCC patients was isolated and sequenced (hereafter referred to as exoRNA). Interestingly, the results of cfRNA module preservation analysis in the exoRNA dataset showed strong evidence of overall preservation (Zsum > 10) of almost all (7/8) tested modules (Figure 3A). Only the cf-yellow cfRNA module had weak to moderate overall preservation (Zsum > 2). As Zsum is a composite statistic, we also visualized its main components—Zdensity (density preservation) and Zconnectivity (connectivity preservation) (Figure 3B). Zdensity describes the interconnectedness of the module nodes in the test network while Zconnectivity denotes the similarity of the connectivity patterns of module nodes in the test network compared with the reference network. The cf-turquoise module from the cfRNA dataset showed strong evidence of density (Zdensity > 10) and connectivity (Zconnectivity > 10) preservation, while most other modules revealed stronger evidence of preservation of the connectivity compared to the density of the modules (Figure 3B). We also tested the module preservation of the exoRNA modules (Supplementary Figure S2B) in the cfRNA dataset by performing the reverse analysis (Supplementary Figure S4). We again observed strong overall preservation (Zsum > 10) of most (4/6) exoRNA modules in the cfRNA dataset, indicating high reproducibility of gene network modules obtained from liquid biopsies of HCC patients (Supplementary Figure S3).

### cfRNA module preservation across transcriptomes of liver cell types

**FIGURE 4:**
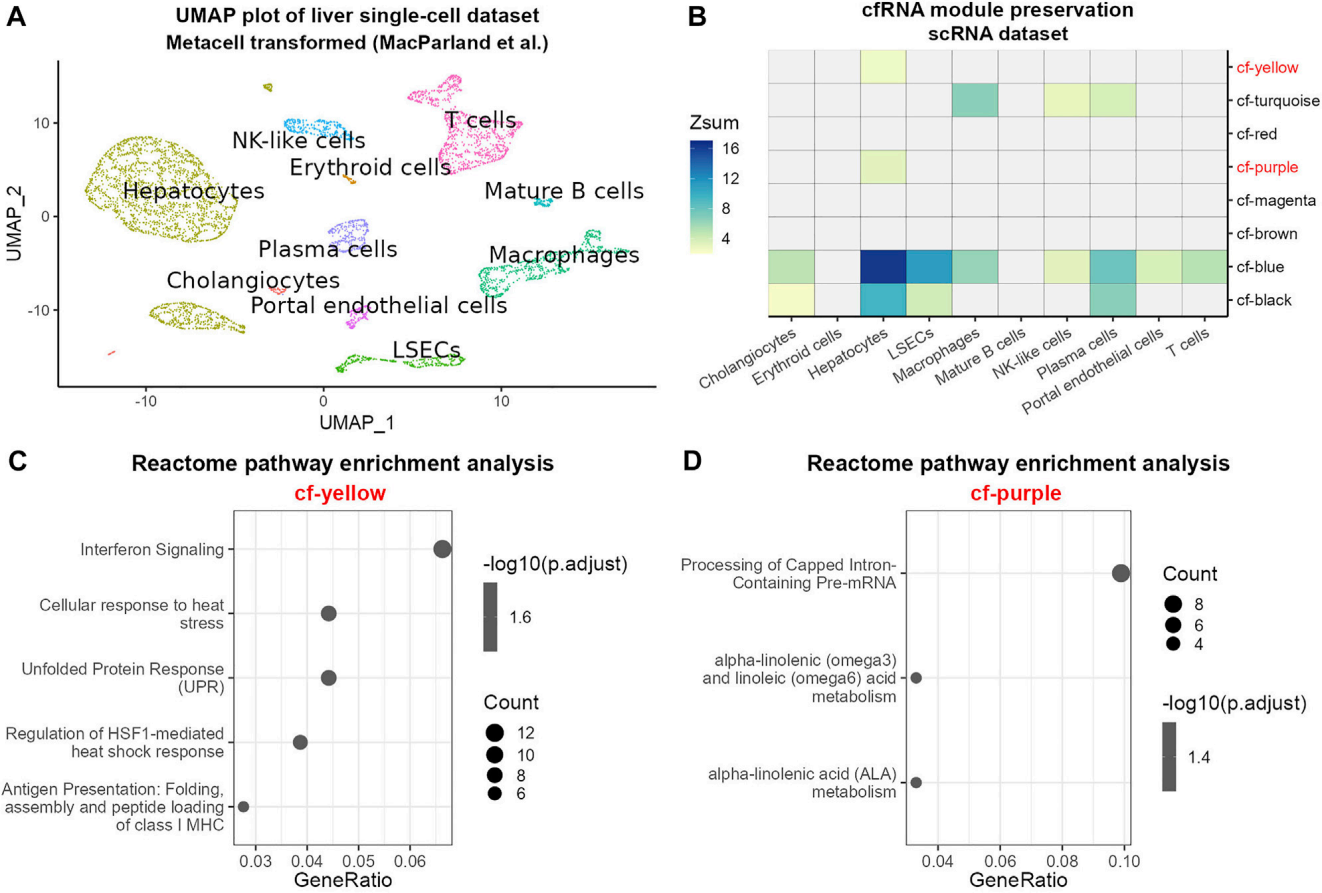
Module preservation analysis of cfRNA modules in scRNA data. **(A)** UMAP plot of scRNA metacells after aggregation of similar cells colored by cell type. **(B)** Heatmap of cfRNA module preservation in cell-type specific transcriptomes; the color scale indicates the value of Zsum preservation statistic. Pathway enrichment analysis of modules cf-yellow **(C)** and cf-purple **(D)**. Point size denotes the number of genes in each pathway; the color scale is proportional to the −log10 transformed adjusted *p*-value; GeneRatio describes the proportion of genes found in each pathway relative to the total number of input genes found in the database.

The composition of cfRNA in the blood represents a pool of RNAs derived from several tissues and cell types (Vorperian et al., 2022). Hence, changes in cfRNA between healthy and HCC patients could reflect transcriptomic changes in several cell types. In order to gain insights into the cellular sources of modules from cfRNA which distinguish between healthy and diseased patients, we aimed at leveraging single-cell sequencing data to test to which extent cfRNA modules are preserved in different cell types of the liver. We chose a dataset containing single-cell sequencing data derived from healthy livers in order to detect cell-type specific transcriptomic changes as a consequence of the disease (Supplementary Figure S6A) (MacParland et al., 2018). Filtering out cell types with an insufficient number of cells (Materials and Methods) resulted in the retention of 10 cell types which were robustly detected in the dataset (Figure 4A). In order to test associations of cfRNA modules, particularly modules displaying strong differentiating ability between healthy and HCC samples, with transcriptomic profiles of cell types found in the human liver, we performed module preservation analysis using modules identified in the previous analysis.

The results of module preservation analysis (Figure 4B) showed strong overall preservation of the modules cf-blue, cf-turquoise and cf-black in several cell types. Interestingly, the module cf-blue, which showed high disease association, was highly preserved in hepatocytes and LSECs. We also noticed that the modules cf-purple and cf-yellow showed preservation exclusively in hepatocytes and hence were analyzed further by pathway enrichment analysis (Figures 4C,D, Supplementary Figure S5). We found an enrichment of pathways related to fatty acid metabolism and mRNA processing in module cf-purple and pathways related to unfolded protein and heat shock response, antigen presentation and interferon signaling in module cf-yellow, indicating cellular stress likely related to pathological alterations of the cells (Figures 4C,D).

In order to validate the module preservation in liver cell types, we conducted the same analysis using exoRNA dataset (Supplementary Figure S6B). Similar to the previously analyzed dataset, we found an exoRNA module displaying high preservation in particular in hepatocytes and LSECs which is also enriched for RNA translation related pathways (Supplementary Figures S6C–E). Thus, the combination of WGCNA with single cell sequencing data was able to detect strong liver-derived signals in the form of network modules with high differentiating ability between healthy and HCC patients and link these to certain cell types found in the liver.

## Discussion

Early diagnostics of HCC is crucial for the management of its progression and liquid biopsy is a promising tool for HCC diagnostics. In order to better characterize HCC liquid biopsy data, we conducted a weighted gene co-expression network analysis (WGCNA). We were able to find modules and genes of interest which could play a role in HCC liquid biopsy diagnostics. In addition, we found that the results of our study were reproducible in a second HCC liquid biopsy data—further indicating the potential of liquid biopsy for robust diagnostics. Furthermore, we augmented our study with HCC liver single-cell RNA sequencing data which augments traditional WGCNA analysis by linking gene network modules to cell-type specific transcriptomes.

Most cfRNA WGCNA modules showed a strong and significant correlation with the disease state of samples, implying that liquid biopsy modules can be quite informative about the health of the donor and the patient. Some modules also displayed weaker and less significant correlations with age and gender, in particular there was an inverse correlation between age and disease state, gender and disease state. This can be explained by the characteristics of the donors and patients that were sampled in the original study (Zhu et al., 2021) wherein the healthy controls had a higher mean age than HCC patients. Similarly, we found that there were more male participants in the HCC group compared to the healthy control group. When we analyzed the most interconnected and highly correlated members of the modules which highly correlated with disease state, we found numerous genes with known association with HCC, including *IMPDH2* (He et al., 2018), *COL24A1* (Yan et al., 2020), *RACK1* (Cao et al., 2019), *SNRPD2* (Liu et al., 2022) and the hub genes *RPL5* and *TLK1* (Segura-Bayona et al., 2020; Ye et al., 2022). A more comprehensive list of such genes can be found in Supplementary Table S1.

Beyond providing lists of genes, WGCNA’s ability to uncover relationships between detected genes is crucial for construction of networks and pathways facilitating insights into biological functions. Pathway enrichment analysis of the modules that correlated most with the disease trait (cf-blue and cf-turquoise modules) revealed enrichment of pathways generally involved with HCC progression. This is exemplified by the enrichment of L13a mediated silencing of ceruloplasmin expression in the cf-blue module. Ceruloplasmin is a protein that is important for iron homeostasis and is mainly secreted into the blood by the liver (Shang et al., 2020). Furthermore, it has been reported to have a protective role in HCC (Shang et al., 2020). It is thus noteworthy that although cfRNA in general mostly contains blood cell derived RNA (Larson et al., 2021), WGCNA analysis is able to detect signals derived from the liver. Module cf-blue was enriched for pathways related to RNA translation which is known to be dysregulated in HCC (Drozdov et al., 2012; Zou et al., 2019). Another noteworthy pathway enriched in the cf-blue module was “Viral mRNA translation” which may point towards hepatitis-B virus (HBV) or hepatitis-C virus (HCV) infection in HCC patients.

The cf-turquoise module was most prominently enriched for pathways involving Rho family GTPases as well as neutrophil degranulation. Rho family GTPases regulate various cellular functions (Xue et al., 2006) and play an important role in HCC carcinogenesis and metastasis (Wang et al., 2013), particularly GTPase *RAC1* (Xue et al., 2006; Yang et al., 2010; Wu et al., 2011; Wang et al., 2013; Bayo et al., 2021). Neutrophil degranulation describes a process in which neutrophils release granules that promote inflammation and immune response (Othman et al., 2021). Neutrophils and their immune activation through degranulation and release of neutrophil extracellular traps (NETs) in the context of HCC is currently an area of intense investigation since neutrophils present a potential therapeutic target in HCC (Tang et al., 2021; Geh et al., 2022). Our data indicate that neutrophils might not only present a promising therapeutic target but also be an important component for cfRNA diagnostics through liquid biopsies.

As there is a high degree of both technical and biological variability between blood liquid biopsy samples, reproducibility of results remains an important consideration (Yeri et al., 2017; Murillo et al., 2019; Geeurickx and Hendrix, 2020; Tong et al., 2020). To that end, we performed module preservation analysis to test if the modules identified in the cfRNA dataset of Zhu et al. can also be identified in the exoRNA dataset of Li et al. The results showed evidence of preservation for all cfRNA modules, most showing strong evidence of module preservation. A similar analysis of exoRNA modules in cfRNA dataset again revealed evidence of module preservation for all exoRNA modules indicating the robustness of WGCNA based cfRNA analysis. However, it is important to note that the number of available cfRNA datasets for HCC is still small at this point. Thus, it will be crucial to extend this analysis towards more datasets in the future.

To gain more insight into the results obtained from the WGCNA analysis, we leveraged the advantages of single-cell RNA sequencing data such as the ability to decipher cell type specific transcriptomes. We conducted a preservation analysis of cfRNA modules in a scRNA sequencing dataset. Our results indicate the highest preservation of cfRNA and exoRNA modules in hepatocytes which can be explained by the high prevalence of hepatocytes in the liver (Bogdanos et al., 2013). Two cfRNA modules also showed preservation only in hepatocytes and one of the modules was enriched for pathways participating in fatty acid metabolism-a well described characteristic of hepatocytes (Alhazzaa et al., 2013; Alves-Bezerra and Cohen, 2017). While the other module was enriched for pathways indicating cellular stress and perhaps pointing towards viral infection, in particular with the hepatitis-B virus (Seo et al., 2018; Li et al., 2019; Baudi et al., 2021)—a known contributor to the development of hepatocellular carcinoma (Maucort-Boulch et al., 2018). Lipid metabolism and particularly fatty acid metabolism dysregulation is a well described characteristic of different cancers, including HCC (Fernández et al., 2020; Hu et al., 2020). Specifically, changes to lipid metabolism pathways involved in fatty acid desaturation or generation of phosphatidylcholine are associated with proliferating hepatocytes in HCC (Hall et al., 2021). Finally, considering that cf-yellow and cf-purple modules showed strong positive and negative correlation with HCC respectively (Figure 1A) it is possible that WGCNA was able to capture bidirectional gene expression changes associated with HCC.

Interestingly we also detected a noticeable preservation signal in liver sinusoidal endothelial cells (LSECs) in both cfRNA and exoRNA modules. In both cases the modules with high LSEC preservation were enriched for pathways relating to RNA translation which was previously described as characteristic of LSECs (MacParland et al., 2018). LSECs play a significant role in HCC progression (Wilkinson et al., 2020; Yang and Zhang, 2021), produce extracellular vesicles (Azparren-Angulo et al., 2021), are very permeable and in direct contact with the bloodstream due to their endothelial nature (Shetty et al., 2018). Hence, while hepatocytes are understandably the main focus of study in HCC, LSECs and the extracellular vesicles they produce can represent a promising cell type for further investigation as their transcriptomic changes in HCC development might hold important diagnostic insights which could be leveraged by liquid biopsy.

A crucial advantage of combining WGCNA with scRNA sequencing is the ability to query liquid biopsy samples without the need to rely on few disease markers which can be lowly expressed and hence not detected in cfRNA. With the growing accuracy and depth of various gene pathway databases, the modules identified by WGCNA will become more informative and specific, thus increasing the future potential of the method for diagnostics. In general, relying on signatures and pathways related to biological functions as identified through WGCNA modules likely leads to more reliable diagnostics compared to marker genes which might be sporadically detected.

In the future, we envision utilization of already widely available scRNA datasets of healthy and malignant tissues facilitating the diagnosis of liquid biopsy samples. Modules can be detected in liquid biopsy samples and later linked to cellular origins through preservation analysis which can then motivate further directed medical examination. Still, much work needs to be done, in order to improve cfRNA detection for diagnostics. In particular, the amount of cancer-derived cfRNA in the blood of patients might represent a potential bottleneck, in particular for solid tumors. Here, technical improvements for higher sensitivity of RNA detection e.g. from the field of scRNA sequencing will likely benefit the cfRNA sequencing field. Moreover, it should be noted that among all solid tissues, liver is one of the most significant cfRNA contributing tissues, suggesting that cfRNA diagnostics might be of particular interest for detection of liver-related diseases (Larson et al., 2021).

In conclusion, in this study we showed cfRNA network analysis provides an additional layer of information which can be obtained through liquid biopsy. The combination of cfRNA and scRNA analysis will further open new avenues of research and our results show the great potential of scRNA sequencing data for both cfRNA and network studies.

## Data Availability

Publicly available datasets were analyzed in this study. This data can be found here: GSE142987, GSE100207, and GSE115469. The computational code used in this study is available at GitHub: https://github.com/aramsafrast/cfwgcna_hcc

## Supplementary material

Supplementary Material S1 Top 50 most connected genes in each cfRNA module ordered by connectivity.

Supplementary Material S2 Top 50 most connected genes in each exoRNA module ordered by connectivity.

Supplementary Material S3 Top five most significant GO Biological Process terms per cfRNA module ordered by adjusted *p* value. For the modules cf-black and cf-magenta fewer than five GO terms passed the significance threshold (*p* < 0.05).

Supplementary Material S4 Top five most significant GO Biological Process terms per exoRNA module ordered by adjusted *p* value. For the modules exo-yellow and exo-red fewer than five GO terms or no GO term passed the significance threshold (*p* < 0.05).
