# Supplementary material for: Network analysis of hepatocellular carcinoma liquid biopsies augmented by single-cell sequencing data
Source: Front Genet. 2022 Aug 25;13:921195. doi: 10.3389/fgene.2022.921195 (PMC9452847; doi:10.3389/fgene.2022.921195)
Supplement: Supplementary file 2 [file DataSheet5.docx]

Supplementary Information


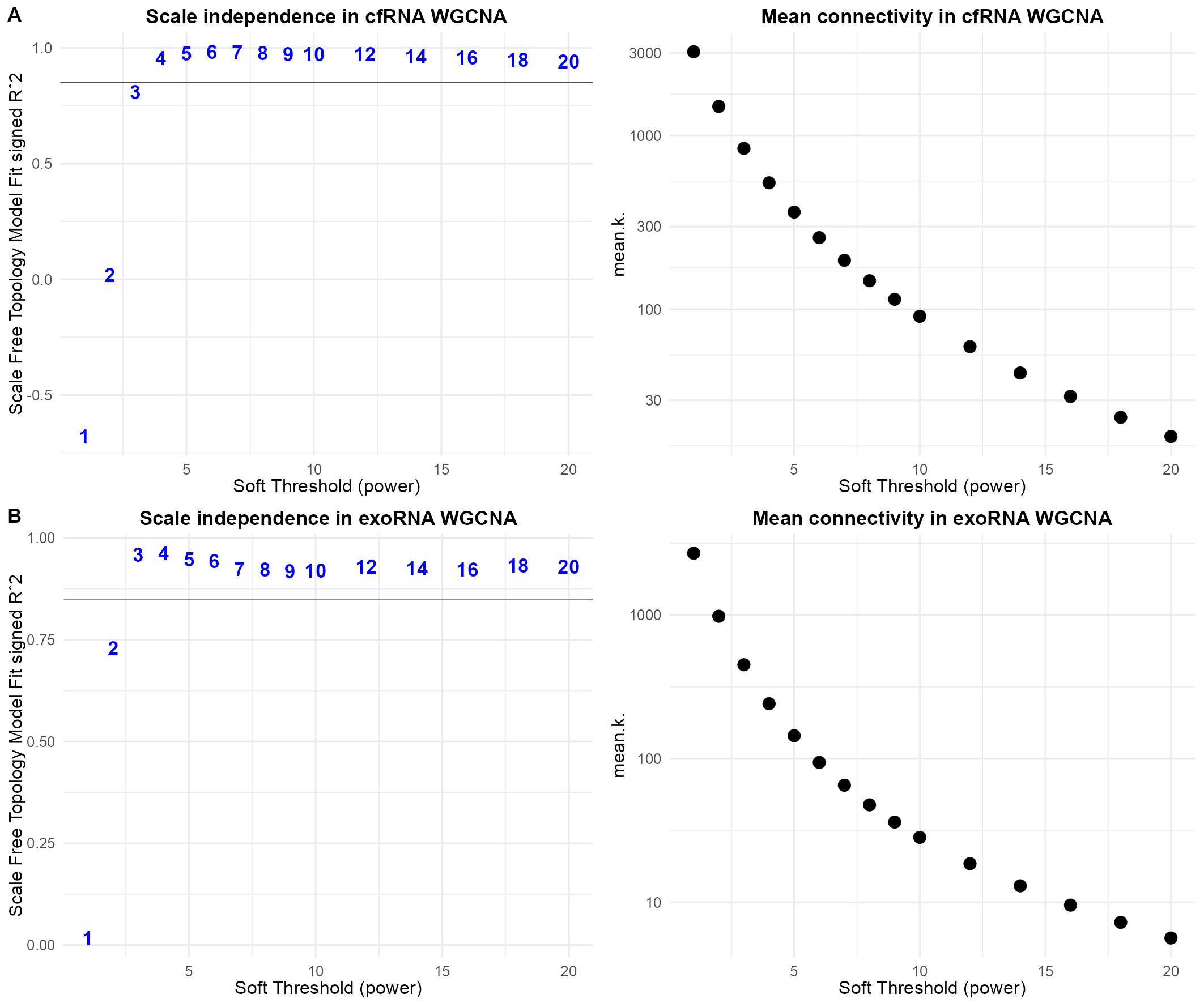


**Supplementary Figure 1**: WGCNA diagnostic plots representing scale free topology fit index and mean connectivity for individual power levels in **(A)** cfRNA, **(B)** exoRNA WGCNA analysis. Horizontal lines represent scale free topology fit index value of 0.8.


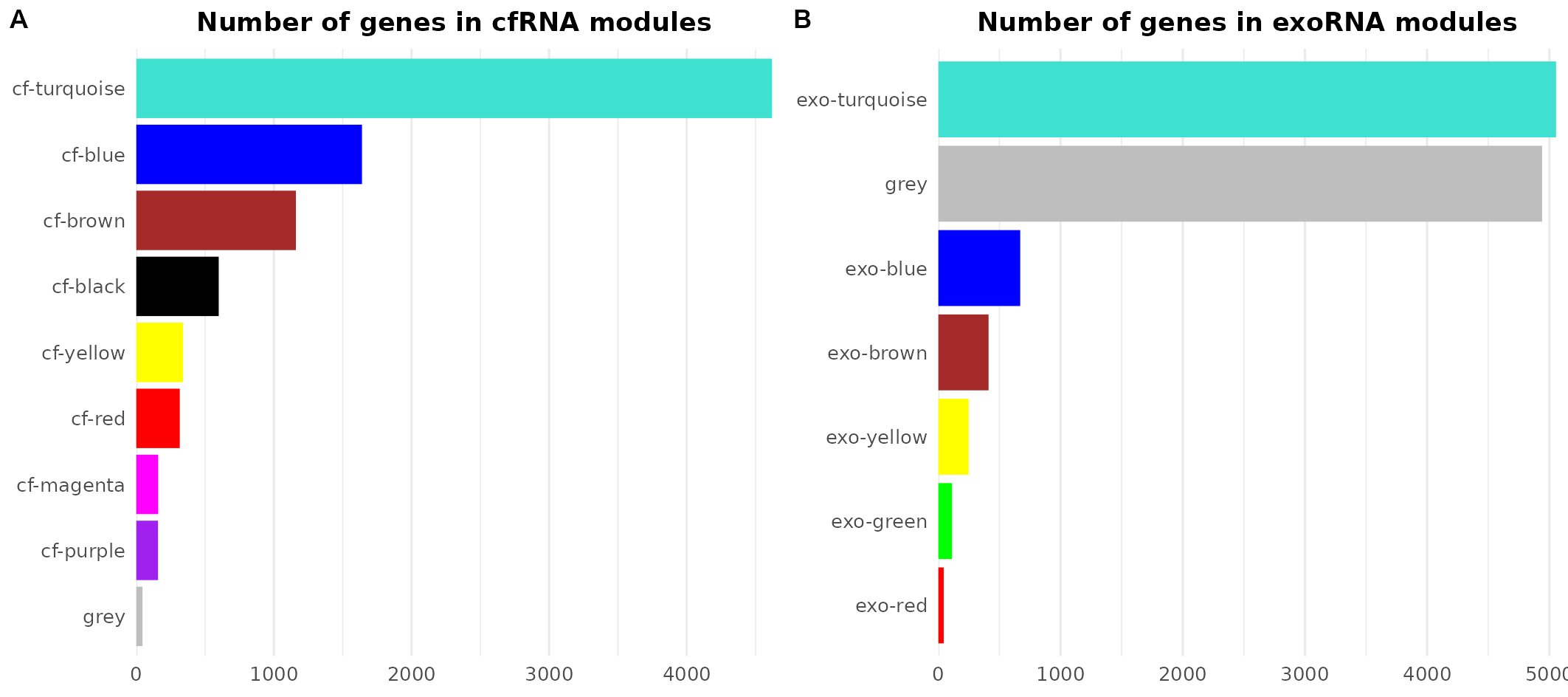


**Supplementary Figure 2**: Number of genes in identified modules for **(A)** cfRNA and **(B)** exoRNA WGCNA analysis. To note, the grey module which is shown here was removed from further analysis.


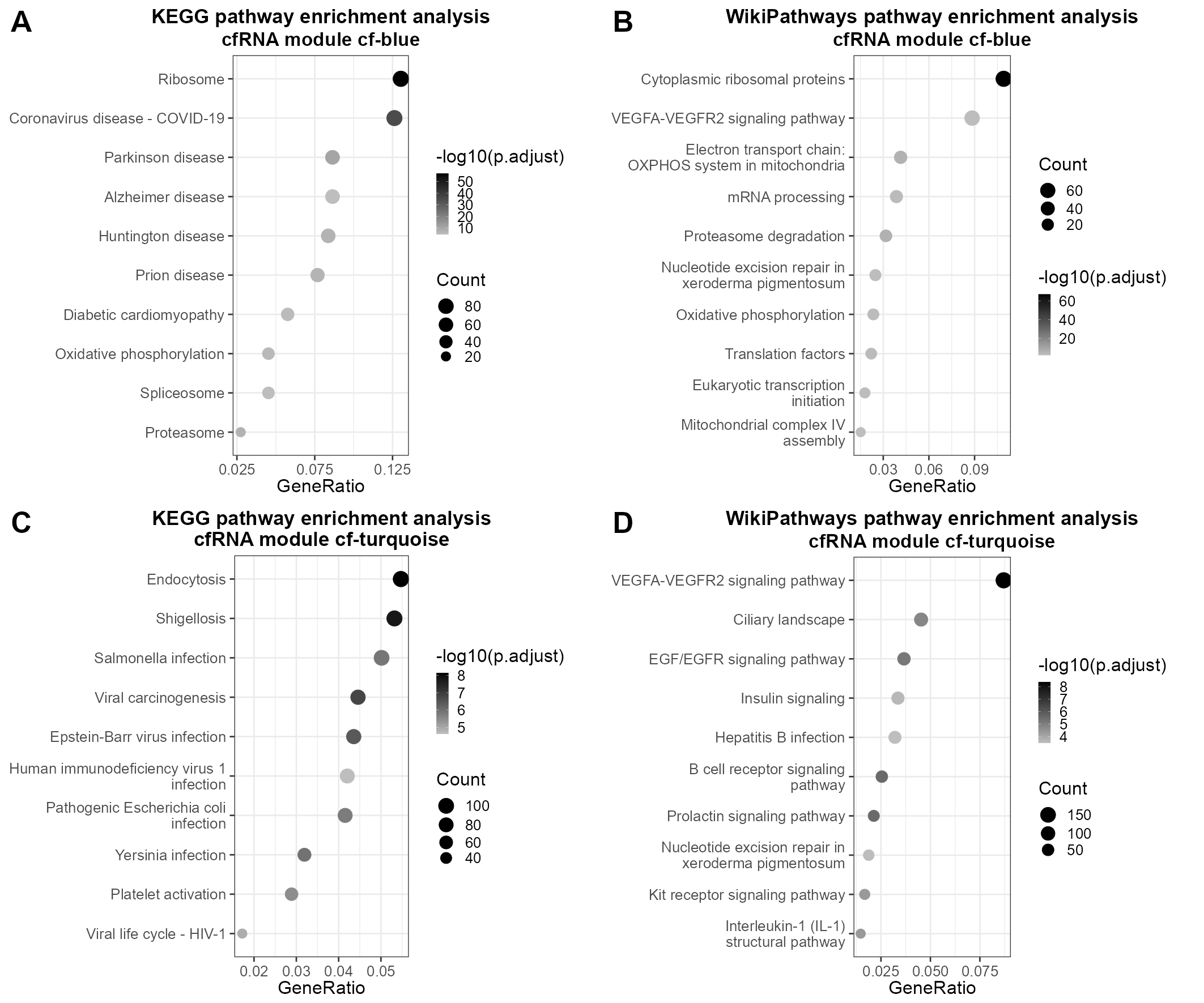


**Supplementary Figure 3**: Pathway enrichment analysis of module cf-blue from the cfRNA dataset with **(A)** KEGG and **(B)** WikiPathways databases and of module cf-turquoise from cfRNA dataset with **(C)** KEGG and **(D)** WikiPathways databases; point size denotes the number of genes in each pathway; the color scale is proportional to the -log10 transformed adjusted p-value; GeneRatio describes the proportion of genes found in each pathway relative to the total number of input genes found in the database.


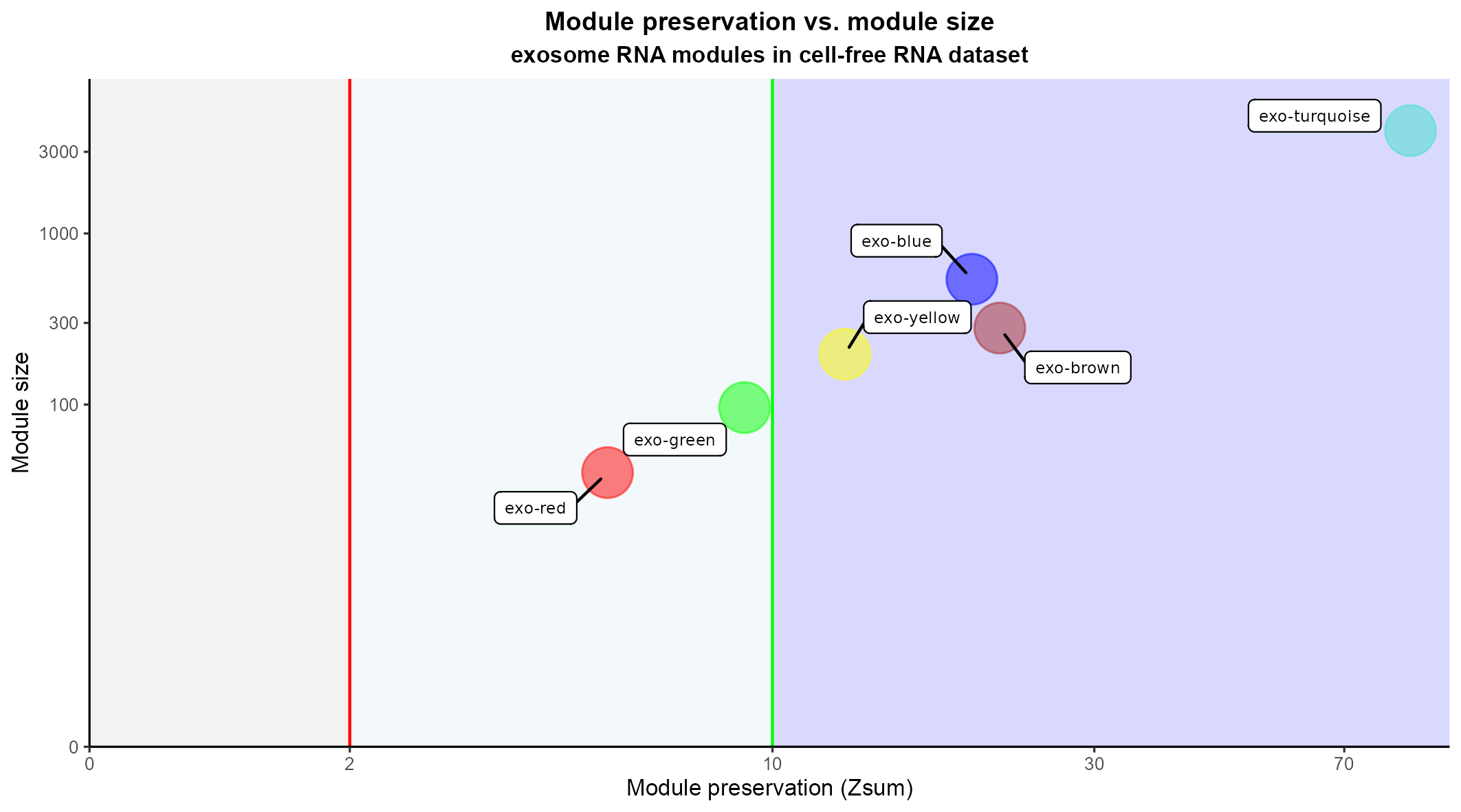


**Supplementary Figure 4**: Overall module preservation (Zsum) of exoRNA modules in cfRNA dataset and its relationship with exoRNA module size. First vertical line represents Zsum=2 and the second vertical line - Zsum=10.  Zsum>2 entails weak to moderate evidence of module preservation, while Zsum>10 denotes strong evidence of module preservation.

**
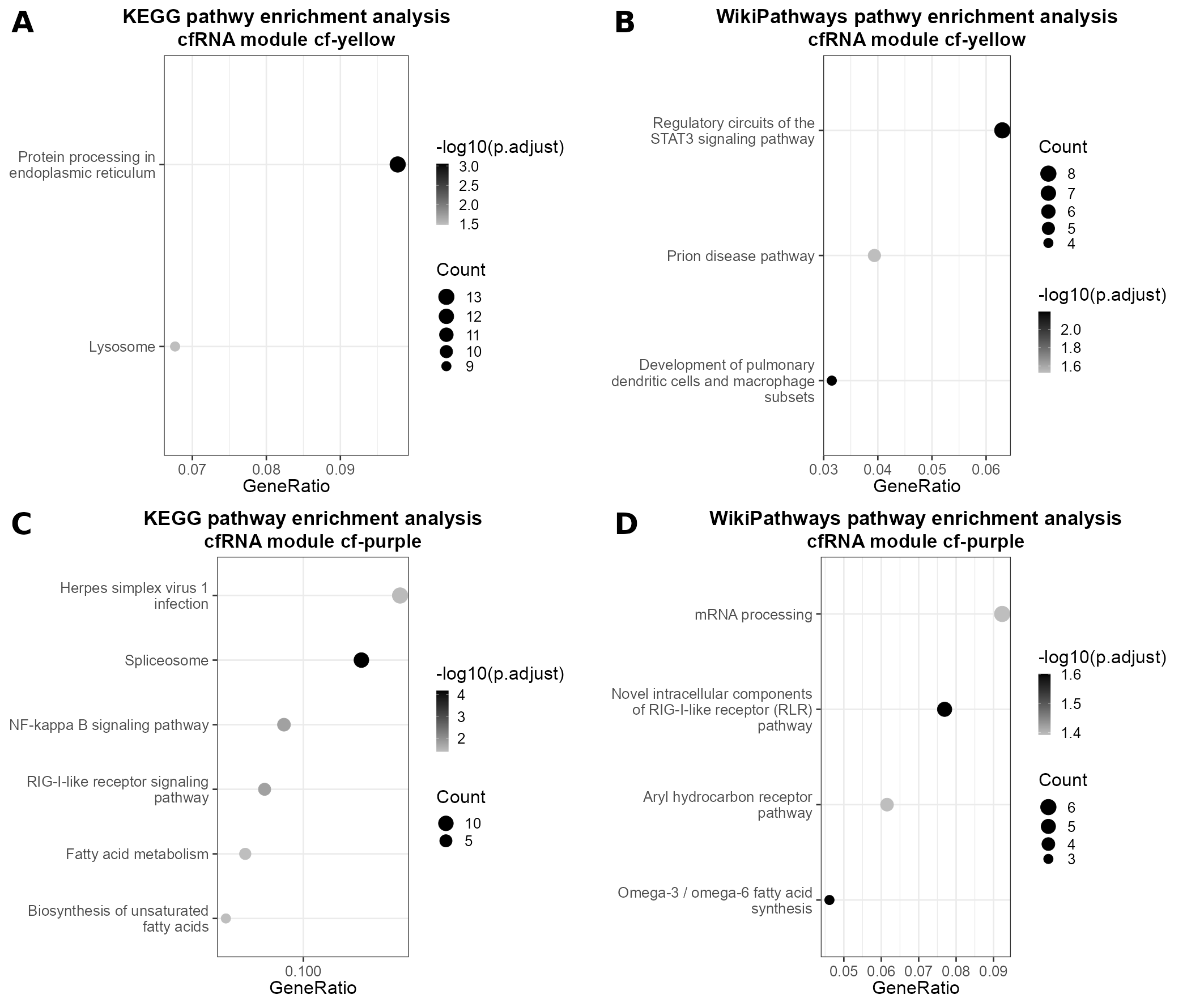
**

**Supplementary Figure 5**: Pathway enrichment analysis of module cf-yellow from the cfRNA dataset with **(A)** KEGG and **(B)** WikiPathways databases and of module cf-purple from cfRNA dataset with **(C)** KEGG and **(D)** WikiPathways databases; point size denotes the number of genes in each pathway; the color scale is proportional to the -log10 transformed adjusted p-value; GeneRatio describes the proportion of genes found in each pathway relative to the total number of input genes found in the database.


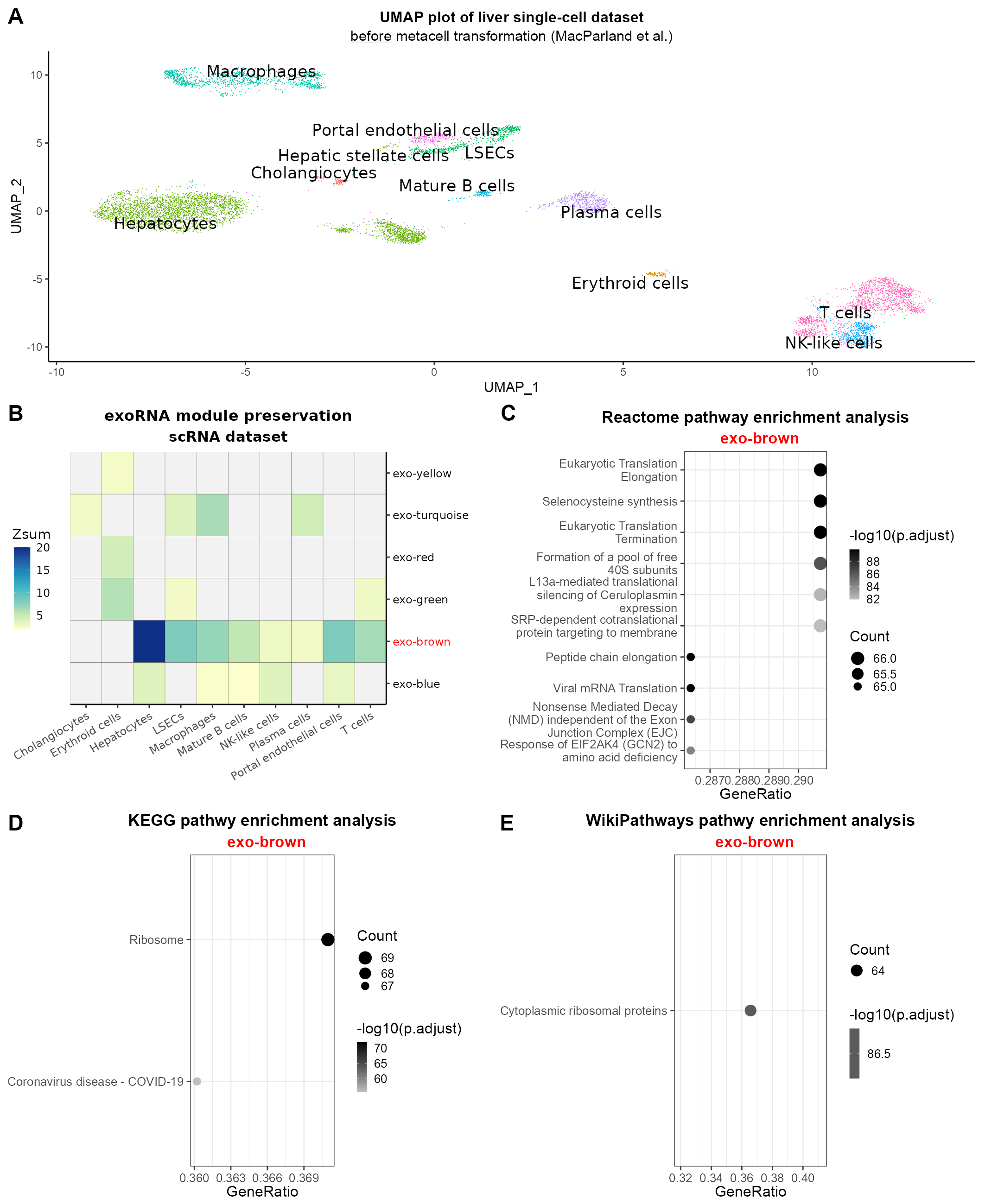


**Supplementary Figure 6**: **(A)** UMAP plot of scRNA dataset before aggregation and colored by cell type. **(B)** Module preservation analysis of exoRNA modules in scRNA cell-type specific datasets; heatmap coloring scale based on Zsum values; “exo_” - modules identified in the exoRNA dataset. Pathway enrichment analysis of module exo-brown from the exoRNA dataset with **(C)** Reactome, **(D)** KEGG and **(E)** WikiPathways databases; point size denotes the number of genes in each pathway; the color scale is proportional to the -log10 transformed adjusted p-value; GeneRatio describes the proportion of genes found in each pathway relative to the total number of input genes found in the database

**Supplementary Table 1**: List of cfRNA cf-blue and cf-turquoise module genes displaying high connectivity and high gene-trait correlation with known association to HCC.

| **Module cf-blue of cfRNA dataset** | **Module cf-turquoise of cfRNA dataset** |
| --- | --- |
| RPSA (Drozdov et al., 2012; Bi et al., 2020) | IMPDH2 (He et al., 2018) |
| EEF1G (Hassan et al., 2018) | SENP3-EIF4A1 (Wang et al., 2020) |
| RACK1 (Li and Xie, 2015; Cao et al., 2019; Xu et al., 2021) | CORO1A (Hu and Gao, 2012) |
| RPL8 (Dolezal et al., 2018) | SNRPD2 (Tu et al., 2019; Gu et al., 2020; Gao et al., 2021; Liu et al., 2022) |
| RPL10A (Zhang et al., 2021) | GTF2F1 (Tu et al., 2019) |
| EEF1B2 (Hassan et al., 2018) | TSC22D1 (Bidkhori et al., 2018) |
| ITPR1 (Fa et al., 2019) | ACRBP (Fu et al., 2015; Luo et al., 2020) |
| ANTXR2 (Xing et al., 2018) | MIR-4435-2HG (Kong et al., 2019) |
| RPN2 (Huang et al., 2019) | UGGT1 (Bidkhori et al., 2018) |
| UBR4 (Kong et al., 2020) | ITPR2 (Bidkhori et al., 2018) |
| COL24A1 (Yan et al., 2020) | APP (Wu et al., 2020) |
| LGMN (Andrade et al., 2011) | NLK (Chen et al., 2015) |
| HNRNPA1 (Zhou et al., 2013) | SSX2IP (Li et al., 2013) |
| RPS3A (Zhou et al., 2020) | YWHAZ (Zhao et al., 2018) |
| RPL14 (Liu et al., 2007) | KIF2A (Liu et al., 2021b) |
| RPS24 (Liu et al., 2007) | LIMS1 (Zou et al., 2021) |
| EIF3H (Zhu et al., 2016) | YWHAE (Liu et al., 2013) |
| RPL5 (Ye et al., 2022) | SNAP23 (Liu et al., 2021a) |
|  | PRUNE1 (Bibbò et al., 2021) |
|  | CLCN3 (Wang et al., 2018) |
|  | ASAP1 (Wang et al., 2016) |
|  | CORO1C (Wang et al., 2013) |
|  | PRDX6 (Xu et al., 2016) |
|  | RAP1B (Tang et al., 2018) |
|  | PTPN12 (Luo et al., 2014) |
|  | TLK1 (Segura-Bayona et al., 2020) |
